# The Family Health Scale: Reliability and Validity of a Short- and Long-Form

**DOI:** 10.3389/fpubh.2020.587125

**Published:** 2020-11-20

**Authors:** AliceAnn Crandall, Nomi S. Weiss-Laxer, Eliza Broadbent, Erin Kramer Holmes, Brianna Michele Magnusson, Lauren Okano, Jerica M. Berge, Michael D. Barnes, Carl Lee Hanson, Blake L. Jones, Len B. Novilla

**Affiliations:** ^1^Department of Public Health, Brigham Young University, Provo, UT, United States; ^2^Department of Family Medicine, Jacobs School of Medicine and Biomedical Sciences, University at Buffalo, Buffalo, NY, United States; ^3^School of Family Life, Brigham Young University, Provo, UT, United States; ^4^Puget Sound Educational Service District, Renton, WA, United States; ^5^Department of Family Medicine and Community Health, University of Minnesota, Minneapolis, MN, United States; ^6^Department of Psychology, Brigham Young University, Provo, UT, United States

**Keywords:** family health, factor analysis, structural equation modeling, psychometrics, depression

## Abstract

Families strongly influence the health of communities and individuals across the life course, but no validated measure of family health exists. The absence of such a measure has limited the examination of family health trends and the intersection of family health with individual and community health. The purpose of this study was to examine the reliability and validity of the Family Health Scale (FHS), creating a multi-factor long-form and a uniform short-form. The primary sample included 1,050 adults recruited from a national quota sample Qualtrics panel. Mplus version 7 was used to analyze the data using a structural equation modeling framework. Exploratory factor analysis (EFA) and confirmatory factor analysis (CFA) confirmed a 32-item, 4-factor long-form scale. The four factors included (1) family social and emotional health processes; (2) family healthy lifestyle; (3) family health resources; and (4) family external social supports. A 10-item short-form of the FHS was also validated in the initial sample and a second sample of 401 adults. Both the long-form and short-form FHS correlated in the expected direction with validated measures of family functioning and healthy lifestyle. A preliminary assessment of clinical cutoffs in the short-form were correlated with depression risk. The FHS offers the potential to assess family health trends and to develop accessible, de-identified databases on the well-being of families. Important next steps include validating the scale among multiple family members and collecting longitudinal data.

## Introduction

The health of individuals and communities begins with the developmental foundations laid in the family (1, 2). The individuals comprising families as well as external environments (e.g., neighborhood, community, society, policy, and law) influence family health routines, norms, and ultimately family health (3). Family health is “a resource at the level of the family unit that develops from the intersection of the health of each family member, their interactions and capacities, as well as the family's physical, social, emotional, economic, and medical resources” (4). Healthy families promote a sense of belonging and build family members' ability to develop, care for each other, and meet life's responsibilities. Family health routines and norms are developed as family members share their understandings, opinions, and behaviors about health and healthcare, which in turn reinforces individual health habits. Families are an unparalleled influence and resource for maintaining health and preventing disease because members may support and nurture one another through the various life stages in ways that no other system can (5, 6). In fact, the economic value of the care that families provide across the life course is far greater than that provided by the medical system (7, 8).

Despite the centrality and economic value of the family in producing health, little attention has been paid to measuring family health, in part because no validated family health measure exists. Public health frameworks, such as Healthy People 2020 (9) and Public Health 3.0 (10), place the individual at the center of community health and neglect the critically important role of families as producers and central contexts for health. In fact, from a review of family health content assessed in six routinely administered U.S. national health surveys, there is a nearly exclusive focus on individual health status of family members, behaviors, and medical care utilization (4). When family measures are included, they typically focus on a narrow range of family sociodemographic factors such as family structure, household composition, income, race, and occasionally individual members' health (11). The near absence of family health and functioning measurement in U.S. population health surveys limits our understanding of the connections between individual, family, and community health. Moreover, this gap powerfully limits the development of public health programs, policies, and goals to strengthen the well-being of American families, to evaluate these interventions, and to understand longitudinal trajectories of family health. Although public health and other agencies recognize the need for these data, no adequate, valid, and reliable measures of family health currently exist.

### Family Health as an Interdisciplinary Concept

To fully understand the health of a family, one must consider multiple factors such as family functioning, communication and problem-solving skills, routines, mental health, emotional support, economic resources, adequate housing, transportation, education, health insurance, healthy eating and physical activity behaviors, adequate childcare, and access to external resources. These factors transcend the disciplinary boundaries of public health, family studies, psychology, social work, medicine, and other disciplines. It is essential to measure these interdisciplinary factors in order to provide a more holistic measure of family health. In addition, an assessment of family health would likely encourage more disciplines to measure this construct, which may ultimately lead to better interventions, program development, health care, educational programs, and other resources for families. Creating a family health scale will also ensure that we are consistently measuring a critical construct of a fundamental unit of society that holds power for increasing or decreasing individual- and population-level health.

### Family Frameworks as a Guide for Measuring Family Health

Some existing scales, such as the Family Assessment Device (12) and the Family Adaptability and Cohesion Evaluation Scales (FACES) Circumplex Model (13), have been used as proxies for family health, but essentially only measure one aspect of family health: family functioning. Although no family health scales exist, there are core features of current family measurement that lend themselves well to a more holistic analysis of family health. First, a family is an organized unit of interdependent individuals. Thus, individual functioning relates to the complex system of behaviors within and between members of the family system (14, 15). This may include routines and rituals that individuals enact within their given family system [e.g., (16, 17)]. It may also include considering bi-directional influences of health such as how one family member's health may affect and shape another family member's health. Second, the family is also composed of interdependent relational subsystems. Hence, assessing the quality of the relationships that an individual experiences within the family is likewise an important part of understanding the family system. Third, scholars such as Bronfenbrenner and Morris (18) add ecological sensitivity to family systems models, emphasizing that families operate within a broader socioeconomic and geopolitical context where resources or demands outside of the family may interact with resources and demands inside the family that impact family functioning (18). Incorporating these assumptions will help improve the understanding of family health.

### Theoretical Foundations of Family Health Measurement

Foundational work to define and measure family health and its core concepts and domains began with an interdisciplinary group of family health experts who formed the Family Health Maternal and Child Health Measurement Resource Network (Family Health MRN), supported by the Maternal and Child Health Bureau of the U.S. Health and Human Services Administration (4). Using a three-round Delphi expert process, the team identified key domains and concepts of family health. Thirty-one family health concepts were generated and organized into six priority domain areas: family relationships, family social context, family member health, family health-related practices, family health resources, and management of time and activities. Authors of the study concluded that future work should develop and operationalize a measure of family health that examined the relevance of the key domains and concepts to multiple family formations.

The Family Health MRN built off of Denham's Family Health Framework (3). Denham's framework was developed from interviews of families in Appalachia. Her three-dimensional framework included functional (e.g., relationships and interactions), contextual (e.g., internal and external contexts such as family socioeconomic status and social supports), and structural (e.g., family routines) aspects of family health. The Denham framework focused on families living in the same household, thereby excluding both extended and immediate family members not living under the same roof.

Both the Family Health MRN and Denham's Family Health Framework provide important contributions to developing a measure of family health. Although the names of the domains/aspects of the two frameworks differ, their concepts are similar. The Family Health MRN's framework, however, is more conducive to the variation and complexity of family types, while Denham's model recognizes the importance of the external contextual environment as relevant to the family's health.

### Purpose of the Study

The lack of a validated multidimensional measure of family health limits the examination of the state and trends of family health in the U.S. and globally. Therefore, the purpose of this study was to develop, refine, validate and test a scale to measure family health that was applicable across disciplines and based on the theoretical foundations of the Family Health MRN and Denham's Family Health Framework. This study included the following aims:

#### Development and Refinement Aims

1) Develop and refine a final long-form version of the FHS (FHS-LF), comprised of ~20–40 items. The FHS-LF will include multiple dimensions of family health as separate subscales, and it will be important for research questions where understanding different aspects of family health is important.
2) Conduct exploratory factor analysis (EFA) to determine the factor structure of the Family Health Scale (FHS) in a national, diverse sample based on socioeconomic status and family type.
3) Develop a short-form of the FHS (FHS-SF) comprised of 8–12 items. The FHS-SF will be a unidimensional measure of family health. It will not have separate subscales for the different dimensions of family health, but the abbreviated number of items will allow for a uniform measure of family health when survey space is limited.

#### Validation Aims

4) Test the validity of the FHS factor structure using confirmatory factor analysis (CFA) in a random split replication sample.
5) Examine the validity of the FHS-LF and FHS-SF by assessing correlations of the FHS-LF subscales and the FHS-SF with validated family measures that assess single dimensions of family health (such as family functioning and healthy lifestyle). We expected moderate to high positive correlations between measures.

#### Testing Aims

6) Examine whether the FHS items are invariant across gender, age, and marital status among adults.
7) Determine clinical cutoffs for family health using the FHS.

**Figure 1:**
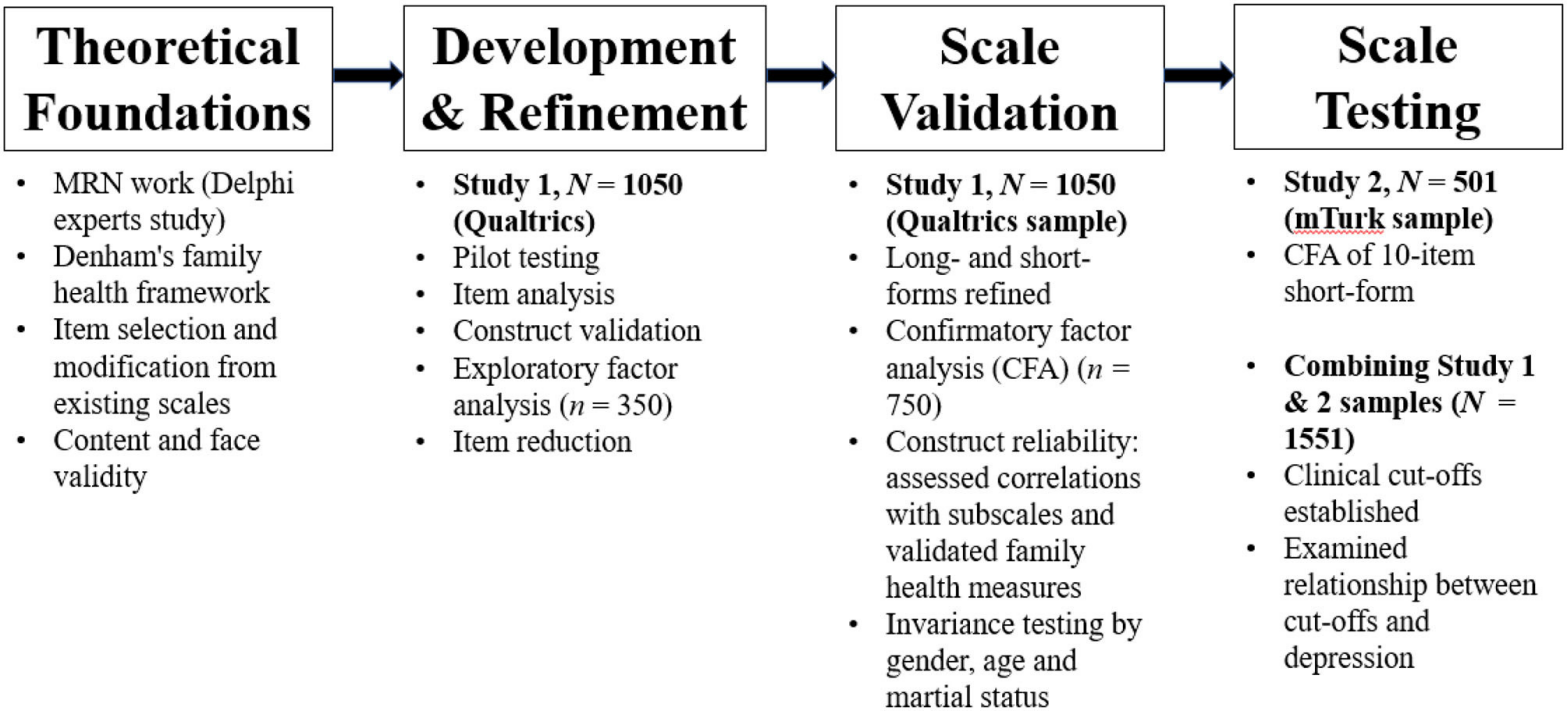
Family health scale development (long- and short-form) methods summary.

To examine the aforementioned aims and guided by the best practices of scale development and validation in the social sciences (19, 20) we conducted two studies described below (see Figure 1 for a summary of Methods and Samples).

## Scale Piloting

### Procedures for Scale Development and Piloting

An interdisciplinary team of 11 family health experts (including the authors of this paper) developed a set of items for a pilot version of the FHS. Team members represented the fields of public health, psychology, family science, and medicine.

#### Pilot Round 1

The team initially developed a set of 67 items that fit the domains and concepts identified by the Family Health MRN. Many items were adapted from questions in previously validated scales [e.g., Family Adaptability and Cohesion Scale (21), Family Cohesion Scale (22), Family Resilience Assessment (23), Family Health Climate Scale (24), Benevolent Childhood Experiences Scale (25), Walsh Family Resilience Questionnaire (26), Chronic Stressors (27), Family Functioning, Health and Social Support Family Functioning Scale (28), Family Relationships Index (29), Conflicts and Problem Solving Scale (30), and the Household Food Insecurity Scale (31)]. The 67 items were piloted among 500 adults from different family types (e.g., married, single, households with children, empty nesters, etc.) recruited via Amazon Mechanical Turk (mTurk). Participants were compensated $2.50 USD for completing the survey. Results from exploratory factor analysis showed a four-factor scale (family social and emotional health processes, family healthy lifestyle, family socioeconomic resources, and family emotional resources).

#### Pilot Round 2

The team made further revisions and piloted the scale again because three of the four factors had very few items after dropping items with low factor loadings or high cross-loadings on more than one factor. Additionally, the team wanted to test items that fit on a factor relating to external supports, identified as an important aspect of family health in Denham's Family Health Framework. The pilot round 2 FHS had 51 items and was administered to a second sample of 500 adults recruited via mTurk who came from different family types (e.g., married, single, households with children, empty nesters, etc.); to increase representativeness in this pilot, a subset of the sample was required to be low income or to have less than a high school diploma. Five factors were identified in this version of the FHS (family social and emotional health processes, family healthy lifestyle, family socioeconomic resources, family help-seeking efficacy, and family external social supports); four items were dropped due to low loadings on any factor or high cross loadings across two or more factors (>|0.30| on two or more factors).

After the round 2 pilot, the team had some concerns that some factors from the second pilot were due to differences in responses options across questions and/or the wording of question stems. Thus, additional revisions were made to the scale to ensure uniformity in response options across items before administering to the final sample.

## Study 1: Measure Development, Factor Analysis, Construct Validity, and Measurement Invariance

### Methods

#### Sample and Procedures

A sample of 1,050 U.S. adults was recruited from a Qualtrics panel. Qualtrics, a software company, actively manages a proprietary panel of members who are recruited for participation in research. Panelists who meet initial screening criteria were invited to participate in the survey, which included the FHS and other scales thought to be correlated with family health. Demographic quotas for low education, minority race or ethnicity, being born outside of the U.S., and family structure (e.g., married, single, households with children, empty nesters, etc.) based on estimates from the U.S. Census were used to ensure a sample that was varied and representative of the U.S. population. Compensation varied, with those comprising harder to recruit demographics being compensated at a higher rate. Compensation for all subjects was < $4.00 USD each. A sample size of 1,050 provided adequate power to conduct EFA and CFA based on the number of factors and items that we anticipated (32). The study was approved by the University Institutional Review Board (IRB).

The sample demographics are provided in Table 1. Broadly, the sample was 53.8% female, 60.9% white, 46.1% married, and 67.7% with a child under 18 living in the home. Single parent households comprised 18.6% of surveyed respondents.

**Table 1:** Demographic characteristics of the sample.

**Demographic**	**Total sample** **(*N* = 1,050)**	**EFA sample** **(*n* = 350)**	**CFA sample** **(*n* = 700)**
Female	53.8	56.0	52.7
Age (M)	40.3	39.9	40.5
Married	46.1	43.7	47.3
Have children	64.8	66.0	64.1
% with child <18	67.7	68.0	67.6
Single parent	18.6	20.0	17.9
< High school education	12.0	16.0	10.0
Employed	60.6	59.7	61.0
Live in single-family home	49.2	47.1	50.3
Race			
White	60.9	59.3	61.6
Black	17.0	13.4	18.8
Asian	6.4	7.3	5.9
Hispanic	11.0	14.2	9.4
Other	4.7	5.8	4.2
Born in U.S.	89.0	89.1	89.0
Annual income < $10,000	14.6	17.8	13.1
Annual income > $180,000	4.8	4.6	4.9
# of people living in household (M)	3.2	3.2	3.2
Live alone	10.0	12.3	8.9

#### Measures

##### Family health

The final FHS included 47 items. Response options across all questions were on a 5-point Likert Scale ranging from Strongly Disagree to Strongly Agree. Negatively worded items were reverse scored so that higher scores indicated better family health.

##### Scales for construct validation

To examine construct reliability of the FHS, we included two scales that measured single aspects of family well-being thought to be similar to specific domains of family health. We included the 13-item Family Functioning subscale from the McMaster Family Assessment Device (FAD) (12), which we felt most clearly aligned with a factor relating to family social and emotional health processes (a factor identified in the pilot phases). Response options for the Family Functioning scale ranged from 1 (strongly disagree) to 4 (strongly agree). The four-item Family Health Climate Score (24) was included and thought to be especially similar to the Family Healthy Lifestyle subscale identified in the pilot. Response options ranged from 1 (definitely false) to 4 (definitely true).

#### Data Analysis

##### Testing reliability of the FHS-LF

EFA and CFA were performed using a structural equation modeling framework in Mplus Version 7. The sample was randomly split using one-third (*n* = 350) for the EFA and two-thirds (*n* = 700) for the CFA (see Table 1 for demographics for the EFA and CFA samples). To ensure appropriate internal reliability of the scale with 20–40 items in the long-form, we employed a fairly high standard for item inclusion: items were sequentially dropped if they had a loading lower than |0.50| or a cross-loading above |0.30|. The same factor loading cut-offs (determined a priori) were examined in the CFA on the two-thirds random split sample.

All models were estimated using a robust weighted least squares estimation, which is appropriate for categorical data like the ordinal response options in the FHS. Full information maximum likelihood (FIML) was used to handle any missing data. Adequate model fit was indicated based on a comparative fit index (CFI) of >0.90 and a root mean square error of approximation (RMSEA) <0.08 (33), theoretical interpretation, and a minimum of three items for each factor.

##### Development of the FHS-SF

After examining the reliability of a long-form, we selected items for the short-form that had high factor loadings and a strong theoretical rationale. Our goal was to develop a valid and reliable short-form with 8-12 items. To assess factor loadings, we included all items that were retained in the FHS-LF in a single factor CFA. We then selected two or three items from each subscale that loaded highly in the single-factor and that also supported different aspects of family health as identified by the Family Health MRN and Denham frameworks. We examined these items in CFA, correlating error terms from items that came from the same subscale. To be retained, we determined a priori that each item had to have a factor loading >0.40.

##### Scale validation

To assess scale construct validity, the subscales (factors) from the long-form were correlated with the latent variables of validated family scales using the CFA random split sample. Likewise, we examined the correlation of the FHS-SF with these same validated scales.

##### Measurement invariance

To test whether the FHS items were invariant across gender (males and females), age (>40 compared to age 18–40 years), and marital status (married and not married) we tested for uniform differential item functioning (DIF) (34, 35). Uniform DIF can be ascertained if at the same levels of family health, men and women (or people older vs. younger; or married vs. not married) score significantly different on individual items and this difference is uniform and consistent across different levels of family health. To test this, we first added gender to the CFA model for the FHS-LF by regressing the FHS-LF subscales on gender and examining the modification indices. A modification index between an FHS item and gender that was >4 suggested uniform DIF for that item. We then controlled for DIF in the model by regressing the FHS item on gender. We followed the same procedures for age and marital status.

### Results

#### Exploratory Factor Analysis

A four-factor model fit the data best (see Supplemental Table 1 in Supplementary Material for item means). Model fit was better with EFA models of 5–7 factors; however, some factors in these models had fewer than the required three items. Model fit was worse for 1–3 factors; additionally, eigenvalues and scree plots best supported a 4-factor model. We dropped 15 items due to low loadings or high cross-loadings. The results of the final four-factor EFA are included in Table 2. Factor 1 contained 13 items and was named “Family Social and Emotional Health Processes.” There were six items in factor 2, which was named “Family Healthy Lifestyle.” Factor 3, “Family Health Resources,” had nine items and factor 4, “Family External Social Supports,” had four items. Cronbach's alphas were high across all four factors (Family Social and Emotional Health Processes: α = 0.92; Family Healthy Lifestyle: α = 0.87; Family Health Resources: α = 0.82; Family External Social Support: α = 0.85).

**Table 2:** Exploratory factor analysis results of the FHS-LF, *n* = 350 (RMSEA = 0.061; CFI = 0.960).

	**Factor 1** **Family social and emotional health processes**	**Factor 2** **Family healthy lifestyle**	**Factor 3** **Family health resources**	**Factor 4** **Family external social supports**
FHS1. We rarely express affection to each other (R).	**0.652**	−0.055	0.098	−0.022
FHS2. There is a feeling of togetherness.	**0.760**	0.056	0.042	0.034
FHS3. We care for one another.	**0.932**	0.000	−0.022	−0.075
FHS4. We support each other.	**0.879**	0.062	0.072	−0.030
FHS5. We rarely do things together (R).	**0.639**	−0.120	0.261	−0.097
FHS6. The things we do for each other make us feel a part of the family.	**0.761**	0.130	−0.061	0.012
FHS7. We have fun together.	**0.817**	0.129	−0.134	−0.011
FHS9. We discuss problems and feel good about the solutions.	**0.683**	0.148	−0.147	0.024
FHS11. Family members pay attention to me.	**0.673**	0.047	0.039	0.194
FHS12. Overall, I am happy with my relationship with my family members.	**0.767**	−0.012	0.032	0.171
FHS13. I feel safe in my family relationships.	**0.796**	−0.033	0.085	0.179
FHS15. We make a point of being physically active during daily life.	0.041	**0.574**	−0.109	0.267
FHS17. We usually have fresh fruits and vegetables in our home.	0.038	**0.696**	0.129	0.025
FHS18. We help each other avoid unhealthy habits.	−0.015	**0.767**	0.010	0.077
FHS19. We make a point to follow medical recommendations.	0.043	**0.775**	0.125	−0.059
FHS20. We help each other in seeking health care services when needed (such as making doctor's appointments).	0.127	**0.689**	0.160	−0.017
FHS21. We help each other make healthy changes.	0.167	**0.739**	−0.056	0.072
FHS23. We stay hopeful even in difficult times.	**0.553**	0.256	−0.010	0.043
FHS25. We have beliefs that give us comfort.	**0.556**	0.167	−0.027	0.072
FHS28. If we needed help from others, we would have real difficulty finding transportation to get to that help (R).	0.146	−0.169	**0.688**	0.162
FHS29. If we needed outside help, we would not know what sort of help was available (R).	0.071	−0.043	**0.647**	0.163
FHS30. Financial difficulties would be an obstacle to getting outside help (R).	0.000	−0.084	**0.756**	0.133
FHS31. We do not trust doctors and other health professionals (R).	0.001	0.244	**0.522**	−0.114
FHS32. A lack of health insurance would prevent us from asking for medical help (e.g., no health insurance or inadequate coverage) (R).	−0.113	0.045	**0.578**	0.025
FHS33. We have people outside of our family who we can turn to for help (such as for advice, help with childcare, a ride somewhere, or to borrow some money or something valuable)?	−0.011	0.043	0.079	**0.753**
FHS34. We have people outside of our family we can turn to when we have problems at school or work.	0.100	−0.008	−0.046	**0.816**
FHS35. If we needed financial help, we have people outside of our family we could turn to for a loan (e.g., for $200)	0.020	0.100	0.120	**0.708**
FHS36. If we needed help, we have people outside of our family who could provide our family with a place to live.	0.000	0.038	0.018	**0.796**
FHS38. My MENTAL health or the MENTAL health of my family members got in the way of MY FAMILY's normal daily activities (such as household chores, work, school, or recreation) (R).	0.103	0.068	**0.503**	−0.161
FHS42. Family worries and problems distracted me when I was working (R).	0.062	0.066	**0.612**	−0.198
FHS43. My family did not have enough money at the end of the month after bills were paid (R).	−0.139	0.197	**0.673**	0.160
FHS47. My family did not have adequate housing (R).	−0.028	0.243	**0.669**	−0.033

*Loadings in bold indicate the final factor/subscale that the factor loaded with*.

#### Confirmatory Factor Analysis

CFA confirmed the results found in the EFA. Model fit was adequate based on the RMSEA (0.059) and good based on the CFI (0.958). Factor loadings ranged from 0.60 to 0.90 for Family Social and Emotional Health Processes, from 0.71 to 0.88 on Family Healthy Lifestyle, from 0.52 to 0.77 for Family Health Resources, and from 0.83 to 0.86 for Family External Social Support (see Table 3).

**Table 3:** Confirmatory factor analysis results of the FHS-LF, *n* = 700 (RMSEA = 0.059; CFI = 0.958).

	**Factor 1** **Family social/emotional health processes**	**Factor 2** **Family healthy lifestyle**	**Factor 3 Family health resources**	**Factor 4** **Family external social supports**
FHS1. We rarely express affection to each other (R).	0.595			
FHS2. There is a feeling of togetherness.	0.803			
FHS3. We care for one another.	0.833			
FHS4. We support each other.^a^	0.866			
FHS5. We rarely do things together (R).	0.658			
FHS6. The things we do for each other make us feel a part of the family.	0.792			
FHS7. We have fun together.	0.862			
FHS9. We discuss problems and feel good about the solutions.	0.763			
FHS11. Family members pay attention to me.	0.798			
FHS12. Overall, I am happy with my relationship with my family members.	0.895			
FHS13. I feel safe in my family relationships.^a^	0.879			
FHS15. We make a point of being physically active during daily life.		0.764		
FHS17. We usually have fresh fruits and vegetables in our home.		0.712		
FHS18. We help each other avoid unhealthy habits.		0.705		
FHS19. We make a point to follow medical recommendations.		0.808		
FHS20. We help each other in seeking health care services when needed (such as making doctor's appointments).^a^		0.828		
FHS21. We help each other make healthy changes.^a^		0.875		
FHS23. We stay hopeful even in difficult times.^a^	0.654			
FHS25. We have beliefs that give us comfort.	0.650			
FHS28. If we needed help from others, we would have real difficulty finding transportation to get to that help (R).			0.668	
FHS29. If we needed outside help, we would not know what sort of help was available (R).			0.722	
FHS30. Financial difficulties would be an obstacle to getting outside help (R).			0.681	
FHS31. We do not trust doctors and other health professionals.^a^ (R)			0.710	
FHS32. A lack of health insurance would prevent us from asking for medical help (e.g., no health insurance or inadequate coverage) (R).			0.521	
FHS33. We have people outside of our family who we can turn to for help (such as for advice, help with childcare, a ride somewhere, or to borrow some money or something valuable)?				0.826
FHS34. We have people outside of our family we can turn to when we have problems at school or work.^a^				0.864
FHS35. If we needed financial help, we have people outside of our family we could turn to for a loan (e.g., for $200).^a^				0.832
FHS36. If we needed help, we have people outside of our family who could provide our family with a place to live.				0.827
FHS38. My MENTAL health or the MENTAL health of my family members got in the way of MY FAMILY's normal daily activities (such as household chores, work, school, or recreation) (R).			0.592	
FHS42. Family worries and problems distracted me when I was working (R).			0.572	
FHS43. My family did not have enough money at the end of the month after bills were paid.^a^ (R)			0.727	
FHS47. My family did not have adequate housing.^a^ (R)			0.765	

a *Included in FHS-SF*.

#### Development of the FHS Short-Form

For the short-form, we selected three items each from the Family Social and Emotional Health Processes and Family Health Resources subscales (the two largest subscales) and two items each from the Family Healthy Lifestyle and Family External Social Supports subscales. The final short-form consisted of 10-items that had adequate model fit based on the RMSEA (0.060) and very good model fit based on the CFI (0.986). Factor loadings ranged from 0.43 to 0.78 (Table 4). Cronbach's alpha for the 10-item scale was 0.80.

**Table 4:** Confirmatory factor analysis results of the FHS-SF, *n* = 350 (RMSEA = 0.060; CFI = 0.986).

**Item**	**Factor loading**
We support each other.	0.725
I feel safe in my family relationships.	0.779
We help each other in seeking health care services when needed (such as making doctor's appointments).	0.755
We help each other make healthy changes.	0.765
We stay hopeful even in difficult times.	0.624
We do *not* trust doctors and other health professionals (R).	0.496
We have people outside of our family we can turn to when we have problems at school or work.	0.493
If we needed financial help, we have people outside of our family we could turn to for a loan (e.g., for $200)	0.481
My family did *not* have enough money at the end of the month after bills were paid (R).	0.431
My family did *not* have adequate housing (R).	0.477

#### Scale Validation

The four subscales of the FHS-LF all correlated in the expected direction with the Family Functioning subscale of the FAD and the Family Health Climate Scale (see Table 5). We expected, and observed, strong correlations in particular between Family Social and Emotional Health Processes with the FAD and between the Family Health Climate Scale with Family Healthy Lifestyles. The FHS-SF also correlated well with the FAD (0.89) and the Family Health Climate Score (0.66). The correlations between all scales were significant at the *p* < 0.001 level.

**Table 5:** Correlation of the FAD and family health climate score with the FHS-LF. Model fit: RMSEA = 0.056; CFI = 0.951.

	**FAD**	**Family health climate score**	**Factor 1: family social/emotional health processes**	**Factor 2: family healthy lifestyle**	**Factor 3: family health resources**	**Factor 4: family external social supports**
FAD	1.00					
Family health climate score	0.45	1.00				
Factor 1: family social/emotional health processes	0.86	0.48	1.00			
Factor 2: family healthy lifestyle	0.65	0.78	0.77	1.00		
Factor 3: family health resources	0.61	0.29	0.52	0.45	1.00	
Factor 4: family external social supports	0.46	0.39	0.47	0.49	0.41	1.00

*p < 0.001 for all correlations. FAD, Family Assessment Device*.

#### Measurement Invariance

##### Gender invariance

Females compared to males reported lower Family Healthy Lifestyle (−0.15, *p* < 0.001) and Family Health Resources (−0.11, *p* < 0.01); females also reported lower scores on the FHS-SF (−0.11, *p* < 0.05). There was no difference by gender on Family Social and Emotional Health Processes nor on Family External Social Supports. Four items had a modification index >4, indicating uniform DIF. At the same underlying level of family health, females compared to males were more likely to report more agreement with “In the past 12 months my family did not have enough money at the end of the month after bills were paid” (FHS-SF item) and “In the past 30 days my mental health or the mental health of my family members got in the way of my family's normal daily activities.” Females were less likely to report agreement with “In my family we rarely express affection to each other” and “In my family we discuss problems and feel good about the solutions.” Once DIF was controlled for, the relationship between gender and Family Health Resources was no longer significant (−0.07, *p* = 0.12). For the FHS-SF, once we controlled for the one item that showed DIF, the relationship between gender and the FHS-SF (-0.09, *p* < 0.10) was also no longer significant. All other items in the FHS were invariant by gender. Table 6 includes results with and without controlling for DIF.

**Table 6:** Demographic factors and family health, with and without controlling for uniform DIF, *n* = 700.

	**Family social and emotional health processes**		**Family healthy lifestyle**		**Family health resources**		**Family external social supports**		**FHS-SF**	
	**Without DIF**	**With DIF**	**Without DIF**	**With DIF**	**Without DIF**	**With DIF**	**Without DIF**	**With DIF**	**Without DIF**	**With DIF**
Female	−0.06	−0.06	−0.15^***^	−0.15^***^	−0.11^**^	−0.07	−0.01	−0.01	−0.11^*^	−0.09
Age > 40 years	0.08^*^	0.10^*^	0.03	0.03	0.20^***^	0.20^***^	−0.09^*^	−0.09^*^	0.06	NA
Marital status: married	0.28^***^	0.28^***^	0.30^***^	0.30^***^	0.21^***^	0.25^***^	0.11^**^	0.15^***^	0.32^***^	NA

* p < 0.05.

** p < 0.01.

*** *p < 0.001*.

##### Age invariance

Participants older than 40 years reported greater Family Social and Emotional Health Resources (0.08, *p* < 0.05) and Family Health Resources (0.20, *p* < 0.001) but lower Family External Social Supports (−0.09, *p* < 0.05) compared to younger participants. Age was not associated with Family Healthy Lifestyle nor with the FHS-SF. There was indication of uniform DIF on one item. Holding family health level constant, participants over the age of 40 were less likely to espouse “In my family we have fun together.” After controlling for uniform DIF, age was still associated with Family Social and Emotional Health Processes, Family Health Resources, and Family External Social Supports.

##### Marital status invariance

Those who reported that they were married had better family health across all four FHS-LF subscales (Family Social and Emotional Health Processes: 0.28, *p* < 0.001; Family Healthy Lifestyle: 0.30, *p* < 0.001; Family Health Resources: 0.21, *p* < 0.001; Family External Social Supports: 0.11, *p* < 0.01), and the FHS-SF (0.32, *p* < 0.001). There was evidence of uniform DIF on four items. Those who were married compared to those who were unmarried were less likely to agree that “In my family we rarely do things together” and “In my family we have people outside of our family who we can turn to for help.” Married participants were more likely to agree that “In my family if we needed help from others, we would have real difficulty finding transportation to get to that help” and “In my family if we needed outside help, we would not know what sort of help was available.” Once we controlled for DIF, being married continued to be associated with all four family health subscales.

## Study 2: Confirmatory Factor Analysis of the FHS-SF

### Methods

#### Sample and Procedures

The second study, also approved by the University institutional review board, included a sample of 501 adults who were ages 18 and older and were born in the U.S. The sample was recruited via Amazon Mechanical Turk (mTurk). As with study 1, participants came from a variety of family types. We required that 15% of the sample had a household income < $25,000, 40% were parents, 20% were married, and the remaining 25% had no restrictions and could be from any family demographic or socioeconomic type. Potential participants who were registered mTurk workers and met the qualifications based on their mTurk profile were able to view a description of the study. Those interested in participating were directed to a Qualtrics survey link. After giving consent, participants completed a 10-min survey. A $2.00 incentive was posted to their mTurk account after completing the survey.

The mean age of participants in study 2 was 41.6 years old; 52.1% were female, 64.1% were married, 68.3% had a Bachelor's degree or higher, 80.4% reported their race as White/Caucasian, and 6.0% had a household income of < $10,000/year. The majority of participants (70.1%) were parents, and 80.3% of participants reported that a child lived in their household.

#### Measures

##### Family health

We included the 10-item FHS-SF that was developed in study 1. As appropriate, items were reverse scored so that higher scores indicated better family health.

#### Data Analysis

We conducted the CFA of the FHS-SF in Mplus version 7 using the same factor loading cutoffs (items with factor loadings <0.40 were dropped) and model fit indices (a minimum CFI of 0.90 and a maximum RMSEA of 0.08) as was used in study 1. We correlated the error terms of items that came from the same subscale in the FHS-LF.

### Results

CFA of the FHS-SF in study 2 confirmed the results of study 1. Model fit of the CFA in study 2 was adequate (RMSEA = 0.067; CFI = 0.987). All items had factor loadings above 0.40 with loadings ranging from 0.46 to 0.87. Cronbach's alpha for the FHS-SF in this sample was 0.84.

## Study 3: Establishment of Clinical cutoffs

### Methods

#### Sample

To provide a larger, more robust sample for calculating the clinical cutoffs, we combined the samples from study 1 (Qualtrics panel) and study 2 (mTurk panel). Participants from both samples had answered questions regarding their family health, depressive symptoms, and demographics. The final combined sample size was 1,551.

#### Measures

##### Family health

We used the 10-item FHS-SF that was developed in study 1 and confirmed in study 2. Responses were reverse coded for negatively worded items so that higher scores indicated better family health.

##### Depression

To help with the examination of clinical cutoffs of the FHS-SF, we included the 9-item Patient Health Questionnaire (PHQ-9) (36). Response options on a 4-point Likert Scale ranged from Not at all to Nearly every day, with higher scores indicating more depressive symptoms. Previous studies have indicated strong internal reliability for the PHQ-9 (α = 0.89) (36). For the purposes of this study, we used the clinical cutoffs score: items were summed and we created a binary variable with total scores of 10 or higher (indicating moderate or high depression) coded as 1 and total scores <10 coded as 0.

#### Data Analysis

Stata 16 was used for data analysis to establish clinical cutoff scores. We created a FHS-SF score by summing the ten items. We examined the distribution and mean FHS-SF scores for participants from studies 1 and 2 to determine whether they were significantly different using the Wilcoxon rank-sum test. Next, we created binary variables for each of the ten FHS-SF items. Responses of 4 or higher (indicating agreement or strong agreement) were scored as 1 and responses lower than 4 (neutrality or disagreement with the statement) received a score of 0. Items were then summed so that each participant could have a final family health score between 0 and 10 points. The distribution of scores was then examined to create clinical cutoffs of poor (<25%), moderate (25 to <75%), and excellent family health (75 percentile or higher). To test the clinical cutoffs, a logistic regression was conducted with depression as the outcome and the family health clinical cutoff variable included as a dummy variable. Given the different recruitment methods across samples, we controlled for participant sample (study 1 = 1, study 2 = 2).

### Results

The mean family health score did not vary across the two samples (*p* = 0.40), with participants from study 1 having a mean score of 40.0 and study 2 participants having a mean score of 40.2. Using the FHS score, across the two studies, participants had an average FHS score of 7.4 (median 8) out of 10 points. For clinical cutoffs, scores of <6 points indicated poor family health, scores of 6–8 indicated moderate family health, and scores of 9 or 10 indicated excellent family health. Controlling for sample, participants with moderate family health compared to poor family health were less likely to experience moderate or severe depression (*OR* = 0.28, *p* < 0.001). Likewise, participants with excellent family health compared to poor family health had lower odds of depression (*OR* = 0.07, *p* < 0.001). Participants with excellent family health compared to moderate family health were also less likely to report moderate or severe depression (*OR* = 0.26, *p* < 0.001) (Table 7). We conducted a sensitivity analysis to examine whether results varied when we added participant socioeconomic controls such as marital status, having children, education, age, race, and employment. The results were substantively the same indicating that family type and family demographics did not bias the relationships. Supplemental Table 2 contains the final FHS-LF, with information about the subscales, the short-form, clinical cutoffs, and scoring information.

**Table 7:** Odds ratios for moderate-to-severe depression based on level of family health.

	**Odds ratio (95% confidence interval)**
Poor family health	[Reference]
Moderate family health	0.28 (0.21–0.38)
Excellent family health	0.07 (0.05–0.10)
Sampling control	
Study 1 qualtrics sample	[Reference]
Study 2 mTurk sample	0.59 (0.45–0.78)

## Discussion

The Family Health Scale was developed in response to the need for a validated measure of family health for use in survey research and as a screening tool in healthcare and other settings. The findings suggest that the long-form and short-form versions of the FHS were reliable and valid in a sample that represented a variety of family types and socioeconomic statuses that were generally representative of the U.S. population. Additionally, we tested and found the FHS-SF to be reliable in a second sample of adults. All of the FHS-LF subscales and the FHS-SF had excellent internal reliability based on the results of the CFA and the Cronbach's alphas. Additionally, the short-form and long-form measures had good correlations in the expected direction with validated scales of different aspects of family health and well-being. The majority of items in the FHS were invariant across gender, age, and marital status, though there was evidence of uniform DIF based on gender, age, and marital status on a few items, which can be controlled for in analyses. Finally, we were able to establish clinical cutoffs for poor, moderate, and excellent family health across two samples, and these clinical cutoff scores of family health were associated with risk for moderate or severe depression. Based on these findings, we present the FHS as a resource for researchers wanting to examine family health in populations, trends in family health, and the intersection between individual, family, and community health.

The FHS offers a comprehensive measure of family health compared to other family well-being scales that measure single and very specific domains of family health. For example, the Family Health Climate Score focuses on physical activity and diet but does not capture other aspects of a healthy family lifestyle measured in the FHS-LF that are relevant to the definition of family health (e.g., healthcare use decisions, overall unhealthy habits). Likewise, the Family Social and Emotional Health Processes subscale includes some items similar to the Family Functioning subscale of the FAD (as demonstrated by the high correlation between the scales). However, the FHS also examines other social and emotional family health processes, such as coping and safety in the family, which were less explicitly assessed in the FAD. In comparison with other family measures, the FHS-LF allows greater specificity in examining various aspects of family health based on theoretical frameworks, while the FHS-SF provides a holistic unidimensional measure of family health.

The correlation between the FHS-SF and the family functioning subscale of the FAD was high, which merits further discussion. The FAD primarily is used in research and clinical applications to screen families experiencing problems and assess change following treatment (12). In contrast, the FHS-SF contains fewer items (10 instead of 13) while also addressing a wider array of family health constructs beyond family functioning. Both instruments have merit, and the application of each depends on the purposes for the assessment.

### Dimensions of Family Health and Effects on Lifelong Individual Health

Each of the four FHS-LF subscales identified in our factor analyses uniquely contributes to family health. As the name implies, the Family Social and Emotional Health Processes subscale measures internal processes relating to connection, communication, emotional safety, satisfaction, and coping within the family context. Family Healthy Lifestyle also addresses internal aspects of family health including healthy behaviors and supporting healthy choices and habits. The Family Health Resources subscale examines both internal and external characteristics of health. Internal resources include individual member health, family worries, socioeconomic resources, and help-seeking efficacy. More external resources include access to resources and a family culture of trust of external resources. Family External Social Supports, as the name suggests, focuses on external social supports that are available to families (e.g., social capital).

Prior research demonstrates the importance of each of the subscales to individual health throughout the life course. Healthy social and emotional processes, such as family connectedness and communication, fosters resilience in childhood and adolescence (37, 38), whereas poor family connectedness and communication are associated with higher psychological stress in adolescence (39). In adulthood, healthy family social and emotional processes are associated with better mental health and physical health and overall well-being such as reduced depression, hypertension, and chronic pain (40–45). A healthy family lifestyle is associated with reduced substance use in adolescence (46–48) and higher levels of physical activity (49).

Beyond a family's internal resources is the access to health resources and external supports. Low family resources increase the likelihood of mental health problems and stress among children and adolescents (50). Deprivation of medical care, food, and clothing is associated with both poorer physical and mental health (51) that increases the likelihood of pre-mature death (52). External family social supports promote resilience among children and reduce psychological distress in high school students (53, 54). Among those 60 and older, greater social capital is associated with higher perceived health (55).

### Use of the Scales in Practice

Both the FHS-LF and FHS-SF are ready for use in survey research, though further research is needed in some areas, which we elaborate on below. The FHS-LF is particularly useful for researchers wanting to explore the antecedents and outcomes of the different aspects of family health. The FHS-SF is useful for when researchers need to economize on items in their survey and/or when only a uniform measure of family health is desired.

Family health trends over time are best assessed through nation-wide surveys, for which we strongly advocate the inclusion of the FHS-SF. Additional research is needed to determine if the FHS-SF is invariant over time and whether a smaller number of items from the FHS can adequately serve as proxy measures of family health in large national public health surveys.

Depending on the healthcare application, both forms of the FHS have important merit as a screener or diagnostic tool. A family health measure is important for healthcare specialists (e.g., doctors, nurses, pediatricians, etc.), acknowledging that a “whole person perspective” on health—one that includes both patients' reported symptoms as well as an assessment of home life and family processes—is critical to disease prevention and health promotion. For healthcare providers to support individual and group level health processes, they must find meaningful ways to assess the many ways families are a key part of health. Unfortunately, health specialists are rarely trained in nuanced family processes research, and those who specialize in family processes receive minimal training in the complexities of health prevention, promotion, or disruption. This means the quality of current measurement within either the health or family studies fields is lacking, without interdisciplinary efforts to bridge these important gaps. The FHS-LF may have value to address family needs to accompany diagnostic and treatment modalities. The FHS-SF may have the most value as an intake screener for patients seeking medical attention at routine office visits, and we have provided preliminary clinical cutoff scores to facilitate this. The scoring of each tool could assist healthcare specialists in treating beyond the symptoms through an awareness of patients' home circumstances. Future research should continue to investigate the utility of the clinical cutoff scores found in the two samples in this analysis by investigating the association between these clinical cutoffs with other health outcomes beyond depression.

## Limitations, Future Research, and Conclusion

The current study validated the FHS among a sample of non-institutionalized adults living in the U.S. Future studies should examine the reliability and validity of the FHS among adolescents and among adults residing outside the U.S. as well as examining differences across regions of the U.S. Additionally, for the FHS to be a true family measure, it would be important to validate the measure among multiple family members such as a married couple and/or an adolescent and their parents. Understanding how multiple members of the same family similarly and differentially report their family health is important to better understanding family health and the utility of the FHS among multiple raters.

There was an indication of measurement non-invariance on a few items based on gender, age, and marital status. We note that on the items where there was an indication of measurement non-invariance that in most cases this did not influence the overall relationships in the results. However, researchers should control for uniform DIF in situations where these demographic characteristics are important to the research questions.

The four subscales of the FHS do not perfectly fit with the domains identified by the Family Health MRN or Denham's Family Health Framework. However, the subscales and items comprehensively cover these two theoretical frameworks of family health and may more clearly demonstrate how these domains are similar or different in actual practice. Versions of the FHS were piloted among multiple populations (two mTurk samples) and then validated in a diverse sample of adults living in the U.S. (the final Qualtrics panel). The FHS-LF and FHS-SF both demonstrate good validity and reliability. However, this does not preclude further scale refinement as further research is conducted about family health. As the first comprehensive and interdisciplinary measure of family health, the FHS allows such research to be initiated.

Finally, the clinical cutoffs established in this study are preliminary and were based on the distributions of the FHS-SF in two distinct samples. We note a strong association between the FHS-SF cutoffs developed in the current study with depressive symptoms, but data is not yet available on the association between the FHS-SF outcomes with other health indicators. Further research is necessary to develop a stronger theoretical rationale for the cutoffs and to confirm the cut-offs established in this study.

Despite the above limitations and need for further research, the current study demonstrates the first steps in identifying a reliable, valid, and comprehensive measure of family health among a diverse sample of adults. The FHS answers the urgent need for a measure of family health that can be used in local samples as well as in nationwide surveys. The FHS may also be an important screener of family health in healthcare settings and in programs that provide services to families. Understanding the antecedents and outcomes of family health is important to better strengthening the health of individuals, communities, and nations.

## Data Availability Statement

The datasets presented in this article are not readily available because Generated Datasets may be made available upon request to the corresponding author after approval from the University IRB. Requests to access the datasets should be directed to AliceAnn Crandall, ali_crandall@byu.edu.

## Ethics Statement

The studies involving human participants were reviewed and approved by Brigham Young University Institutional Review Board. The patients/participants provided implied consent to participate in this study.

## Author Contributions

All authors contributed to the development of the scale and surveys, interpretation of results, writing of the manuscript, and approval of the final manuscript. AC oversaw and conceived of the study, conducted the data analysis, and oversaw the writing of the manuscript. NW-L was involved in the conceptualization of the measure and helped to write the Introduction section. EB helped to project manage the development of the scale and survey administration and wrote sections of the Discussion. EH provided theoretical expertise in the development of the measure and helped write sections of the Introduction and Discussion. BM provided methodological expertise and helped to write sections of the Methods. LO was involved in the initial conceptualization of the measure and editing of the manuscript. JB wrote sections of the Introduction. MB, CH, BJ, and LN wrote sections of the Discussion.

## Conflict of Interest

The authors declare that the research was conducted in the absence of any commercial or financial relationships that could be construed as a potential conflict of interest.
