# Supplementary material for: The Family Health Scale: Reliability and Validity of a Short- and Long-Form
Source: Front Public Health. 2020 Nov 20;8:587125. doi: 10.3389/fpubh.2020.587125 (PMC7717993; doi:10.3389/fpubh.2020.587125)
Supplement: Supplementary file 1 [file Table_1.pdf]

Supplemental Table 1. Item means for the total sample, EFA sample, and CFA sample (response codes ranged from 1-5)

Item	Total Sample (N = 1,050)	EFA Sample (n = 350)	CFA Sample (n = 700)
**In my family…**			
1. We rarely express affection to each other. (R)	3.9	3.9	4.0
2. There is a feeling of togetherness.	4.1	4.1	4.2
3. We care for one another.	4.5	4.5	4.6
4. We support each other.	4.4	4.3	4.5
5. We rarely do things together. (R)	3.7	3.7	3.8
6. The things we do for each other make us feel a part of the family.	4.2	4.1	4.2
7. We have fun together.	4.3	4.2	4.3
8. We have a hard time making decisions about how to solve everyday problems. (R) [a]	3.5	3.3	3.6
9. We discuss problems and feel good about the solutions.	3.8	3.8	3.8
10. Sometimes it is hard to be honest and direct with each other. (R) [a]	3.1	3.0	3.1
11. Family members pay attention to me.	3.9	3.9	4.0
12. Overall, I am happy with my relationship with my family members.	4.1	4.1	4.2
13. I feel safe in my family relationships.	4.4	4.3	4.4
14. We maintain a healthy lifestyle. [b]	3.9	3.9	3.9

Item	Total Sample (N = 1,050)	EFA Sample (n = 350)	CFA Sample (n = 700)
15. We make a point of being physically active during daily life.	3.6	3.5	3.6
16. We normally eat a healthy diet. [b]	3.5	3.4	3.5
17. We usually have fresh fruits and vegetables in our home.	4.0	4.0	4.0
18. We help each other avoid unhealthy habits.	3.5	3.5	3.4
19. We make a point to follow medical recommendations.	4.0	4.0	4.0
20. We help each other in seeking health care services when needed (such as making doctor's appointments).	4.1	4.1	4.1
21. We help each other make healthy changes.	3.9	3.9	3.9
22. One or more people in my family use cigarettes, cigars, or pipe tobacco on a daily basis. (R) [a]	3.4	3.5	3.3
23. We stay hopeful even in difficult times.	4.1	4.1	4.2
24. We have strengths to draw on when we face problems. [b]	4.2	4.1	4.2
25. We have beliefs that give us comfort.	4.1	4.1	4.2
26. We spend time in religious/spiritual activities (such as praying, meditating, or reading religious books). [a]	3.3	3.3	3.3
27. We can trust in the help of relatives, friends, neighbors, and/or the community. [b]	3.8	3.7	3.8
28. If we needed help from others, we would have real difficulty finding transportation to get to that help. (R)	3.7	3.6	3.7
29. If we needed outside help, we would not know what sort of help was available. (R)	3.6	3.5	3.6

Item	Total Sample (*N* = 1,050)	EFA Sample (*n* = 350)	CFA Sample (*n* = 700)
30. Financial difficulties would be an obstacle to getting outside help. (R)	3.2	3.0	3.3
31. We do <u>not</u> trust doctors and other health professionals. (R)	4.1	4.1	4.2
32. A lack of health insurance would prevent us from asking for medical help (e.g., no health insurance or inadequate coverage). (R)	3.4	3.4	3.5
33. We have people outside of our family who we can turn to for help (such as for advice, help with childcare, a ride somewhere, or to borrow some money or something valuable)?	3.7	3.6	3.7
34. We have people outside of our family we can turn to when we have problems at school or work.	3.6	3.6	3.6
35. If we needed financial help, we have people outside of our family we could turn to for a loan (e.g., for $200)	3.4	3.3	3.4
36. If we needed help, we have people outside of our family who could provide our family with a place to live.	3.4	3.4	3.4
**In the past 30 days…**			
37. My PHYSICAL health or the PHYSICAL health of my family members got in the way of MY FAMILY's normal daily activities (such as household chores, work, school, or recreation). (R) [b]	3.5	3.5	3.6

Item	Total Sample (N = 1,050)	EFA Sample (n = 350)	CFA Sample (n = 700)
38. My MENTAL health or the MENTAL health of my family members got in the way of MY FAMILY's normal daily activities (such as household chores, work, school, or recreation). (R)	3.6	3.5	3.6
39. My family members and I had frequent minor disagreements (e.g., "spats," getting on each other's nerves). (R) [a]	3.1	3.1	3.1
40. My family members and I had frequent major disagreements (e.g., big fights, "blow-ups"). (R) [a]	3.8	3.8	3.8
41. My family could always afford to eat nutritious meals. [a]	3.8	3.7	3.9
42. Family worries and problems distracted me when I was working. (R)	3.4	3.3	3.4
**In the past 12 months…**			
43. My family did not have enough money at the end of the month after bills were paid. (R)	3.6	3.5	3.7
44. My family had difficulty paying our bills. (R) [b]	3.6	3.5	3.6
45. My family had to eat a limited variety of foods because of a lack of resources. (R) [b]	3.8	3.7	3.8
46. My family had to eat fewer meals in a day because there was not enough food. (R) [b]	4.1	4.0	4.1
47. My family did not have adequate housing. (R)	4.3	4.3	4.3
