# Supplementary material for: The Family Health Scale: Reliability and Validity of a Short- and Long-Form
Source: Front Public Health. 2020 Nov 20;8:587125. doi: 10.3389/fpubh.2020.587125 (PMC7717993; doi:10.3389/fpubh.2020.587125)
Supplement: Supplementary file 2 [file Table_2.pdf]

Family Health Scale – Long Form (FHS-LF)

Instructions: Please indicate how much you agree or disagree that the statements below describe your family. Answer these questions based on <u>who you consider to be your family</u>.

	**Strongly Disagree (1)**	**Somewhat Disagree (2)**	**Neither Agree nor Disagree (3)**	**Somewhat Agree (4)**	**Strongly Agree (5)**
**In my family…**					
1. We rarely express affection to each other.					
2. There is a feeling of togetherness.					
3. We care for one another.					
4. We support each other.					
5. We rarely do things together.					
6. The things we do for each other make us feel a part of the family.					
7. We have fun together.					
8. We discuss problems and feel good about the solutions.					
9. Family members pay attention to me.					
10. Overall, I am happy with my relationship with my family members.					
11. I feel safe in my family relationships.					
12. We make a point of being physically active during daily life.					

Crandall et al. (2020)

	Strongly Disagree (1)	Somewhat Disagree (2)	Neither Agree nor Disagree (3)	Somewhat Agree (4)	Strongly Agree (5)
13. We usually have fresh fruits and vegetables in our home.					
14. We help each other avoid unhealthy habits.					
15. We make a point to follow medical recommendations.					
16. We help each other in seeking health care services when needed (such as making doctor's appointments).					
17. We help each other make healthy changes.					
18. We stay hopeful even in difficult times.					
19. We have beliefs that give us comfort.					
20. If we needed help from others, we would have real difficulty finding transportation to get to that help.					
21. If we needed outside help, we would not know what sort of help was available.					
22. Financial difficulties would be an obstacle to getting outside help.					
23. We do not trust doctors and other health professionals					

	Strongly Disagree (1)	Somewhat Disagree (2)	Neither Agree nor Disagree (3)	Somewhat Agree (4)	Strongly Agree (5)
24. A lack of health insurance would prevent us from asking for medical help (e.g., no health insurance or inadequate coverage).					
25. We have people outside of our family who we can turn to for help (such as for advice, help with childcare, a ride somewhere, or to borrow some money or something valuable)?					
26. We have people outside of our family we can turn to when we have problems at school or work.					
27. If we needed financial help, we have people outside of our family we could turn to for a loan (e.g., for $200)					
28. If we needed help, we have people outside of our family who could provide our family with a place to live.					
**In the past 30 days…**					
29. My MENTAL health or the MENTAL health of my family members got in the way of MY FAMILY's normal daily activities (such as household chores, work, school, or recreation).					

	Strongly Disagree (1)	Somewhat Disagree (2)	Neither Agree nor Disagree (3)	Somewhat Agree (4)	Strongly Agree (5)
30. Family worries and problems distracted me when I was working.					
**In the past 12 months…**					
31. My family did <u>not</u> have enough money at the end of the month after bills were paid.					
32. My family did <u>not</u> have adequate housing.					

<u>Scoring Information</u>
Reverse code the following items: 1, 5, 20-24, 29-32

Family Health Scale Long-form Subscales
Factor 1: Family Social and Emotional Health Processes
Items: 1-11, 18, 19

Factor 2: Family Healthy Lifestyle
Items: 12-17

Factor 3: Family Health Resources
Items: 20-24, 29-32

Factor 4: Family External Social Supports
Items: 25-28

Family Health Scale Short-form
Items: 4, 11, 16, 17, 18, 23, 26, 27, 31, 32

Clinical cutoffs for the short-form

After reverse coding items 23, 31, and 32, create binary variables for each item by coding as “1” items with a score of 4 or 5 and coding as “0” those with a score less than 4. Sum the scores. Poor family health = 0-5 points; moderate family health = 6-8 points; excellent family health = 9-10 points.

To cite: Crandall, A., Weiss-Laxer, N.S., Broadbent, E., Holmes, E., Magnusson, B.M., Okano, L., Berge, J., Barnes, M.D., Hanson, C.L., Jones, B., & Novilla, L.B. (2020). The Family health scale: reliability and validity of a short- and long-form. *Frontiers in Public Health*. doi: 10.3389/fpubh.2020.587125
